# Flexible Epidermal Sensor Power Systems: Innovations in Multidimensional Materials and Biomedical Applications

**DOI:** 10.3390/s25103177

**Published:** 2025-05-18

**Authors:** Sheng Zhang, Shulan Zhou, Zhaotao He, Oresegun Olakunle Ibrahim, Chen Liu, Mengwei Wu, Chunge Wang, Qianqian Wang

**Affiliations:** 1 Ningbo Global Innovation Center, Zhejiang University, Ningbo 315100, China; szhang1984@zju.edu.cn (S.Z.); 22260404@zju.edu.cn (Z.H.);; 2 State Key Laboratory of Fluid Power and Mechatronic Systems, School of Mechanical Engineering, Zhejiang University, Hangzhou 310027, China; 3 Faculty of Science and Engineering, University of Nottingham Ningbo China, Ningbo 315100, China; 4 School of Biological and Chemical Engineering, Ningbo Tech University, Ningbo 315100, China; 5 Polytechnic Institute, Zhejiang University, Hangzhou 310015, China; 6 School of Mechanical and Energy Engineering, Ningbo Tech University, Ningbo 315100, China; wangchunge@nit.zju.edu.cn

**Keywords:** epidermal sensors, flexible power supply, innovative materials, wearable, biomedical application

## Abstract

Epidermal sensors are pivotal components of next-generation wearable technologies. They offer transformative potential in health monitoring, motion tracking, and biomedical applications. This potential stems from their ultra-thin design, skin compatibility, and ability to continuously detect physiological signals. The long-term functionality relies on advanced power systems balancing flexibility, energy density, and environmental resilience. This review highlights four key power strategies: chemical batteries, biofuel cells, environmental energy harvesters, and wireless power transfer. Breakthroughs in multidimensional materials address challenges in ion transport, catalytic stability, and mechanical durability. Structural innovations mitigate issues like dendrite growth and enzyme degradation. These systems enable applications spanning biomarker analysis, motion sensing, and environmental monitoring. By integrating these advancements, this review concludes with a prospective outlook on future directions for epidermal sensor power systems.

## 1. Introduction

In recent years, flexible wearable electronics have demonstrated immense application potential in health monitoring, motion tracking, human–machine interaction, and environmental sensing. Among these, epidermal sensors have emerged as core components of next-generation wearable technologies due to their ultra-thin profile, lightweight nature, high flexibility, and near-perfect mechanical compatibility with human skin [1]. Compared to conventional rigid or textile-based sensors, epidermal sensors can conform directly to the skin surface, enabling non-invasive and continuous physiological signal detection (e.g., sweat composition [2], ECG signals [3,4], and body temperature [5]) while minimizing motion artifact interference, thereby significantly enhancing wear comfort and data reliability [6]. However, the long-term stable operation of such devices critically depends on efficient and sustainable power supply systems. Current power supply approaches largely constrain the performance and broader application of flexible epidermal sensors. A stable, efficient, and compatible power system is essential to ensure accurate and continuous sensor operation while fully realizing their functional potential.

Traditional rigid batteries present integration challenges due to their bulkiness, heavy weight, and poor mechanical flexibility, conflicting with the miniaturization demands of epidermal devices. Meanwhile, external power solutions (e.g., wired connections) compromise wearability and freedom of movement [6]. An ideal power supply for flexible epidermal sensors must meet multiple criteria: excellent flexibility and conformability to adapt to complex body contours and dynamic movements; high energy density to deliver sustained power under strict spatial and weight constraints; and characteristics of safety, environmental friendliness, and cost-effectiveness to enable mass production and practical applications. Consequently, developing flexible power technologies compatible with epidermal sensors has become a pivotal challenge in advancing their real-world implementation.

This review comprehensively summarizes recent advances in power supply systems and associated materials for flexible epidermal sensors [7]. Four primary powering strategies are analyzed: Chemical batteries employing flexible zinc-ion, sodium-ion, and other emerging battery technologies, which provide stable energy through high-density storage units, suitable for long-term continuous monitoring applications such as.

Biofuel cells that harness biocatalytic reactions to directly convert human metabolites into electricity, enabling integrated sensing and power generation for dynamic metabolic monitoring (e.g., glucose and lactate detection) [8,9,10]. Environmental energy harvesting systems that scavenge human mechanical energy or hydrovoltaic transpiration energy, offering maintenance-free solutions for low-power intermittent sensing scenarios [11]. Battery-free wireless power represented by near-field communication (NFC) technology, which utilizes Ag nanowire spiral antennas and rectifier circuits for on-demand wireless energy transfer, eliminating energy storage units while requiring improvements in transmission distance and deformation compatibility [12,13].

Optimization strategies through material innovations are discussed, covering advanced material systems such as MXene (transition metal carbide/nitride) heterojunctions and liquid metal electrodes [14,15], alongside structural designs like bioinspired microporous substrates [16], multimodal encapsulation architectures [17], and system integration. These advancements provide theoretical foundations and technical perspectives for realizing practical applications of next-generation epidermal sensing systems.

## 2. Structural Architecture and Material Considerations for Epidermal Sensor Power Systems

The power supply system for epidermal sensors requires efficient energy conversion, storage, and transmission within limited dimensions, with its core lying in the synergistic design of multilayer heterogeneous structures. A typical system comprises a bioadaptive substrate layer, energy conversion/storage functional layer, flexible interconnect layer, and encapsulation protective layer. The selection of materials and structures for each layer directly determines power efficiency, wearing comfort, and environmental adaptability.

**Figure 1:**
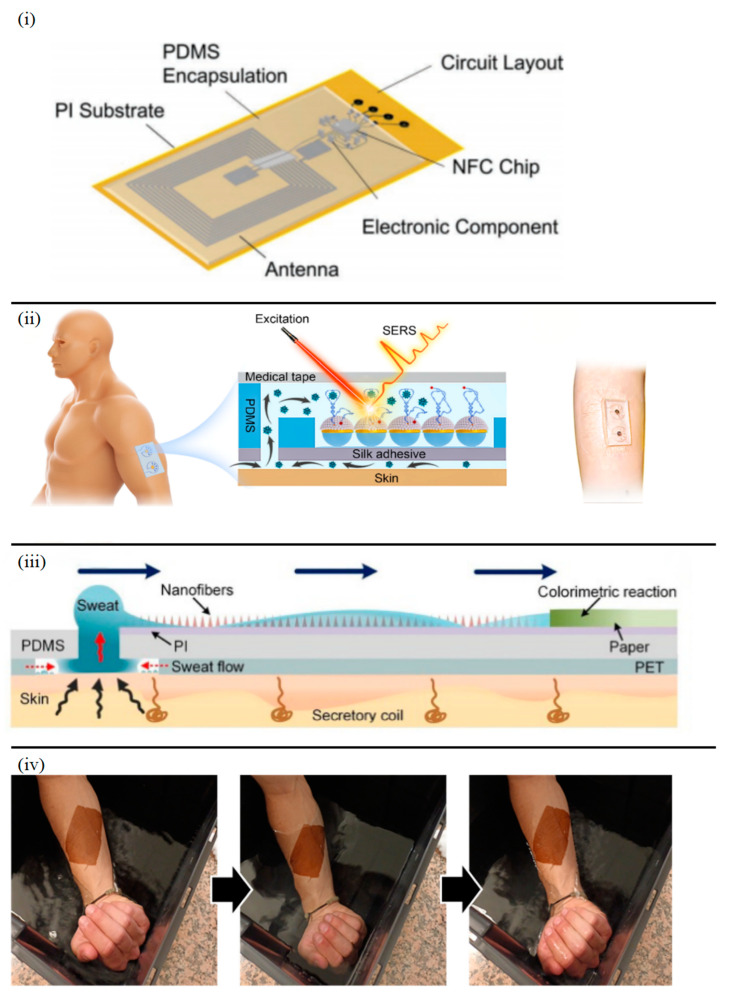
(**i**) PDMS-based bioadaptive substrate for NFC-powered systems [19]; (**ii**) Water-resistant adhesion was demonstrated by subsequent cycles of water immersion of a membrane adhered directly to the skin of a volunteer [12]; (**iii**) Silk fibroin-based biocompatible substrate for cortisol-sensing SERS patch. The red arrow shows secretion from skin. Blue arrows indicate directional transport through the microfluidic channel, guiding sweat to the colorimetric reaction area. This directional flow is driven by the wetting contrast between the hydrophobic PDMS and hydrophilic nanofiber layer [21]; (**iv**) Hydrophilic/hydrophobic bilayer bioadaptive substrate for directional sweat transport [23].

The bioadaptive substrate layer, serving as the direct skin interface, must balance biocompatibility, mechanical flexibility, and environmental responsiveness. While hydrophobic materials like polydimethylsiloxane (PDMS) remain prevalent in chemical batteries and NFC systems for sweat isolation (Figure 1i), recent advancements highlight diversified material strategies [18,19,20,21]. A breakthrough emerges from mussel-inspired bio-adhesive membranes (Figure 1ii), which achieve water-resistant skin adhesion through catecholamine-based polymerization. These membranes maintain >95% cell viability with ultra-conformability, enabling direct NFC sensor integration while resisting sweat interference through controlled hydrophobicity [12].

The team from Southeast University pioneered silk fibroin (SF)-based substrates (Figure 1iii), leveraging its exceptional biocompatibility (>95% cell viability) and mechanical stability to develop a microfluidic surface-enhanced Raman scattering (SERS) patch for cortisol monitoring, demonstrating 85% reduced skin irritation compared to PDMS during 8 h wear [21].

For enhanced sweat management, Wu et al. engineered a Janus-structured hybrid material combining Zn-Al layered double hydroxide (LDH)-modified cotton with laser-engraved medical adhesive, achieving unidirectional sweat transport (11.6 µL cm^−2^ min^−1^) and 100% bacterial inhibition through mechanical “nanoknife” effects [22].

For the bioadaptive substrate layer in epidermal sweat sensing systems, Wang et al. implemented superhydrophobic surface modification on PDMS while fabricating a superhydrophilic nanofiber-enriched microfluidic channel on a polyimide (PI) substrate. This engineered wetting contrast enables directional sweat transport through the synergistic driving of Laplace pressure at the superhydrophobic inlet and capillary forces in the nanofiber microchannel (Figure 1iv) [23], effectively addressing directional control challenges in epidermal sweat collection.

For subdermal applications, Sharma et al. developed dissolvable polycarbonate microneedle arrays (400 µm length) that synergize painless penetration with electrochemical sensing capabilities for continuous interstitial fluid (ISF) biomarker detection [24]. These innovations collectively demonstrate the field’s progression towards hybrid architectures—exemplified by Profusa’s hydrogel-integrated systems—that combine synthetic durability with bioactive interfaces for optimized epidermal compatibility and environmental resilience.

The energy conversion/storage functional layer constitutes the system’s core, with designs varying by power generation mechanisms. In zinc-ion battery systems, the combination of MnO_2_ @MXSC cathodes and three-dimensional porous zinc anodes achieves a specific capacity of 368 mAh/g [25], though zinc dendrite growth remains a challenge requiring electrolyte modification. Biofuel cells employing biocatalytic electrodes like nanoporous Au (NPG) and NPG coated with Pt nanoparticles (PtNPs@NPG) composites demonstrate glucose sensitivity up to 62.33 µA mM^−1^ cm^2^, yet enzyme activity degradation necessitates development of enzyme-free catalytic materials [16].

Flexible interconnect layers perform dual functions of power transmission and mechanical deformation adaptation. While Ag nanowires and liquid metals dominate as conductive materials, their insufficient interfacial adhesion often leads to fatigue failure. Material innovations show distinct dimensional advantages: One-dimensional (1D) nanomaterials like Ag nanowires have become ideal flexible electrode materials through their high transmittance (>90%) and low sheet resistance (<20 Ω sq^−1^) [26]. Two-dimensional MXene-based composites achieve remarkable energy storage performance, as evidenced by the nitrogen-doped mesoporous carbon/MXene hybrid (NMC@MXene-30) showing a specific surface area of 1015 m^2^ g^−1^ and gravimetric capacitance of 393 F g^−1^ at 1 A g^−1^ in aqueous electrolytes. This represents a 240% enhancement compared to pristine MXene (298.52 m^2^ g^−1^) [25,27].

## 3. Power Supply Strategies for Epidermal Sensors

**Figure 2:**
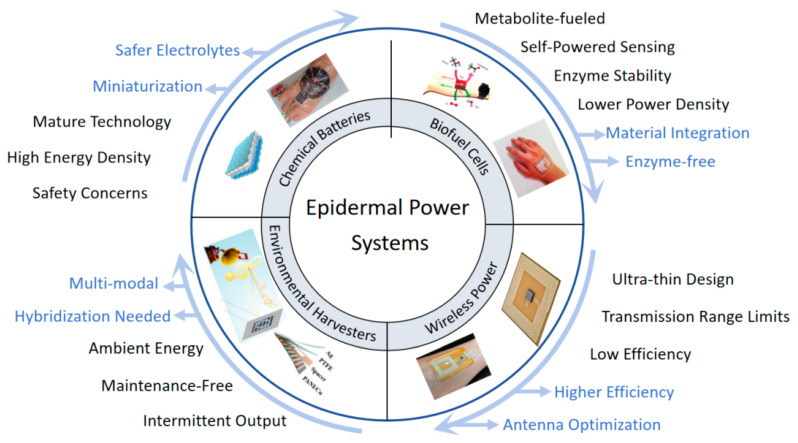
Overview of four primary power supply strategies for epidermal sensor systems. The central theme highlights “Epidermal Power Systems”, branching into four quadrants: Chemical Batteries, Biofuel Cells, Wireless Power, and Environmental Harvesters. Each quadrant includes illustrative examples and key characteristics. Terms in black describe current features and challenges associated with each strategy. Terms in blue, indicated by arrows, represent key development trends and future research directions for advancing these power technologies for epidermal applications.

The preceding discussion has outlined the fundamental structural architecture and key material considerations that underpin the design of power systems for epidermal sensors, focusing on aspects like bioadaptive substrates, functional layers for energy conversion/storage, flexible interconnects, and encapsulation. While these architectural elements are common, the actual energy generation or delivery mechanism employed can vary significantly, leading to diverse performance characteristics and application suitability. Four primary strategies have emerged as particularly prominent for powering the current and next generation of epidermal electronics: Chemical Batteries, Biofuel Cells, Environmental Energy Harvesters, and Wireless Power Transfer. A comparative overview highlighting the core features, inherent challenges, and significant development trends for each of these approaches in the context of epidermal applications is presented (Figure 2). The following subsections will now delve into each of these distinct power supply strategies, examining their operational principles, recent material and structural innovations, specific advantages and limitations, and their overall potential for various epidermal sensing scenarios.

### 3.1. Chemically Powered Epidermal Sensors

Chemical batteries provide high energy density and long-term stability for continuous monitoring applications such as human motion detection. However, safety concerns related to electrolyte leakage and dendrite formation remain critical challenges requiring material-level solutions [28]. Chemically powered epidermal sensors can be categorized into zinc-based batteries, lithium-based batteries, other metal-based batteries, and non-metal batteries based on charge carrier types. The integration of nanomaterials across different dimensions—through optimized electrolyte structures, enhanced interfacial compatibility, and improved ion transport efficiency—has significantly advanced the flexibility, energy density, and long-term stability of these batteries, offering diverse solutions for powering epidermal sensor devices [29,30,31].

#### 3.1.1. Zinc-Based Batteries

**Figure 3:**
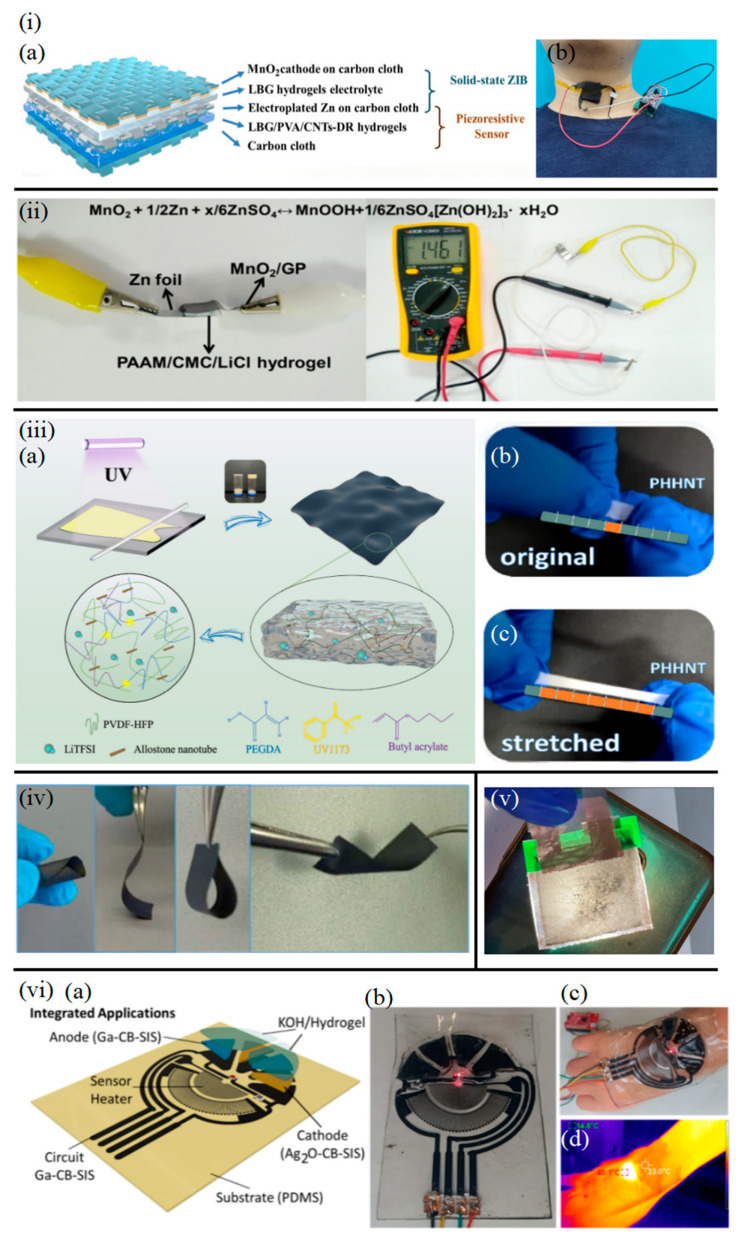
(**ia**) Schematic illustrations of the active powering sensor integrated with a solid-state ZIB in the layer-by-layer view. (**ib**) Photograph of the active powering sensor with a solid-state ZIB mounted on the throat to monitor pronunciation [36]. (**ii**) Photographs of the battery (PAAM/CMC/LiCl hydrogel-based) and the demonstration of its output voltage [44]. (**iiia**) Preparation process and structure diagram of flexible polymer PHHNT electrolyte. (**iiib**) Photograph of PHHNT before stretching. (**iiic**) Photograph of PHHNT after stretching [49]. (**iv**) Photographs of EG-LTO electrodes under different states [52]. Note the colored ruler placed beneath the sample in (**iiib**,**iiic**). This ruler serves as a visual guide to demonstrate the remarkable stretchability of the PHHNT electrolyte. (**v**) Digital images demonstrating the semi-transparency of “all-in-one” battery membrane with flashlight [62]. (**via**) Schematic of layered architecture (Anode: Ga-CB-SIS; Cathode: Ag_2_O-CB-SIS; Substrate: PDMS). (**vib**) Photograph of the circular device with wire connections. (**vic**) Handheld operation under bending. (**vid**) Thermographic image showing heating uniformity (40.1 °C vs. 23.0 °C) [15].

Zinc-based batteries have emerged as a research hotspot for flexible energy storage due to their high safety, low cost, and environmental friendliness [32,33,34]. The incorporation of 1D nanomaterials further enhances their electrochemical performance [35]. Compared to traditional liquid electrolytes, the hydrogel electrolyte developed by Huang et al. [36] offers improved flexibility and safety by mitigating leakage risks, crucial for on-skin applications. The composite hydrogel electrolyte they developed using locust bean gum (LBG) as the matrix. Carbon nanotubes (CNTs) were incorporated to construct a conductive network, achieving a threefold increase in conductivity (0.8 S m^−1^). Coupled with a MnO_2_/CNTs composite anode, the zinc-ion battery retained 98% capacity retention under 30% strain. The 1D structure of CNTs not only enhances electron transport but also suppresses crack propagation in the hydrogel through physical entanglement, addressing the challenge of short cycle life in flexible devices [37]. The integrated flexible zinc-ion battery and pressure sensor system, comprising a solid-state zinc-ion battery (ZIB) and an LBG/polyvinyl alcohol (PVA)/CNTs-DR hydrogel, features five distinct layers, including commercial carbon cloth and natural polymer-based hydrogel layers (Figure 3(ia)). This system demonstrated functionality in detecting human vocalization (Figure 3(ib)).

Lei et al. [38] innovatively designed a zinc-copper galvanic cell using 2D layered double hydroxides (LDHs) to modify electrode surfaces. The 2D channels of LDHs selectively adsorb Zn^2+^ while repelling byproducts, enabling the battery to maintain a coulombic efficiency of 99.2% after 1000 bending cycles, providing reliable energy for high-precision motion sensing.

The introduction of 3D hydrogel networks endows zinc batteries with exceptional mechanical flexibility and self-healing capabilities [39,40,41,42,43]. For instance, Li et al. [44] developed a zinc-ion battery based on a polyacrylamide/carboxymethyl chitosan/LiCl (PAAM/CMC/LiCl) ionic hydrogel (Figure 3ii). By incorporating cross-linker N,N′-methylenebisacrylamide (MBAA) to form a 3D cross-linked network, the hydrogel achieved a tensile strain of 600% and self-healing efficiency (>90%). Paired with a zinc foil anode and MnO_2_/graphite cathode, the battery maintained stable output under bending, compression, and sub-zero temperatures (−20 °C). Its closed-loop system enabled real-time monitoring of finger bending and other human activities via resistance-to-voltage conversion (Figure 3ii), demonstrating the adaptability of 3D materials in dynamic deformation scenarios.

#### 3.1.2. Lithium-Based Batteries

Lithium-based batteries, with their high energy density, are central to wearable devices [45,46]. Performance optimization relies on synergistic design of multidimensional nanomaterials and interfacial engineering innovations. Advances in ion transport efficiency, mechanical flexibility, and integrated design have provided versatile solutions for epidermal sensor power systems.

1D nanotube structures are promising for directional ion transport [47,48]. Zhu et al. [49] developed a UV-cured polyvinylidene fluoride-co-hexafluoropropylene (PVDF-HFP) gel electrolyte with halloysite nanotubes (HNTs) (Figure 3(iiia)). The HNTs’ charged surfaces created Li^+^ transport channels, achieving 1.40 × 10^−3^ S cm^−1^ conductivity. Under mechanical stress, the PHHNT electrolyte stretched to seven times its original length (Figure 3(iiib)). Combined with the 3D network of poly(butyl acrylate-co-ethylene glycol dimethacrylate) (P(BA-co-EGDMA)) copolymer (420% elongation at break), the electrolyte exhibited no shrinkage under 200 °C thermal shock. The assembled LiFePO_4_(LFP) battery demonstrated 89% capacity retention after 120 cycles at 0.5 C. While achieving impressive stretchability (seven times original length) and thermal stability (no shrinkage at 200 °C), the practical challenge for widespread adoption of Li-metal-based flexible batteries remains the mitigation of Li dendrite growth, even with advanced electrolytes, to ensure long-term safety and cycle life.

2D materials offer new avenues for electrode–electrolyte interface optimization [50,51]. Wang et al. [52] developed a layer-interlocked graphene–metal oxide heterostructure electrode. Electrochemically exfoliated graphene (EG) was co-assembled with Li_4_Ti_5_O_12_ (LTO) nanoparticles, forming a 2D/1D composite structure. The 2D EG sheets (lateral size: 1–10 µm) formed a continuous conductive network via π–π stacking, yielding a low interfacial resistance of 143 Ω sq^−1^. The EG-LTO electrode exhibited excellent bending stability, with only an 8% resistance increase after 500 bends (Figure 3iv). A full flexible cell (EG-LTO//EG-LiFePO_4_) retained 98% capacity after 100 cycles at 1 C.

3D porous structures and dynamic cross-linking enhance mechanical adaptability [53,54,55]. Wang et al. [56] synthesized a PEGMEA-ionic liquid/lithium salt composite electrolyte via photopolymerization. Ionic-dipolar interactions between C=O-rich polymer chains and imidazolium cations formed a dynamically reconstructable 3D phase-separated network, endowing the electrolyte with ultra-high stretchability (5.303%). Integrated with silver nanowire (AgNW)-transferred electrodes, the flexible lithium-ion battery maintained a low areal resistance (0.9 Ω^−1^) under 100% strain and >90% capacity retention after 200 cycles, suitable for large joint movements.

Akhmetova et al. [57] pioneered a one-step electrospinning method to co-spin PVDF-HFP nanofibers (diameter ≈ 500 nm) with active materials, creating an “all-in-one” sandwich-structured flexible battery. The 3D nanofiber network provided continuous ion/electron transport pathways, delivering an areal capacity of 5 μAh cm^−2^ with <5% capacity loss after 40 bends. Notably, the design achieved 40% transparency (Figure 3v), laying the groundwork for powering transparent epidermal sensors. The ‘all-in-one’ sandwich structure fabricated by Akhmetova et al. [57] via a single electrospinning step simplifies the battery assembly process compared to multilayer lamination techniques typically used for flexible batteries. The resulting 40% transparency also opens possibilities for less obtrusive or even transparent epidermal power sources, a niche not easily addressed by opaque battery chemistries.

#### 3.1.3. Other Metal-Based Batteries

Gallium-based batteries, with their unique flexibility, high conductivity, and biocompatibility, are emerging for wearable applications [58]. A breakthrough study demonstrated a fully 3D-printed stretchable gallium–carbon composite battery [15,59]. Parvini et al. [15] Developed the anode, made of gallium–carbon black-styrene isoprene copolymer, maintained high conductivity (initial resistance~4 Ω) with 100% strain tolerance. The cathode utilized a silver oxide composite system, integrated with an acrylamide-sodium alginate hydrogel electrolyte and thermoplastic polyurethane (TPU) encapsulation, forming an ultra-thin, waterproof structure. The battery delivered a high areal capacity of 26.86 mAh cm^−2^, with stable performance under stretching and folding. Remarkably, capacity increased by~5% under 50% strain due to liquid metal-induced conductive network optimization, retaining >90% capacity after 80 cycles. The fully 3D-printed stretchable gallium–carbon composite battery by Parvini et al. [15] showcases the unique advantages of liquid metals in achieving both high conductivity and extreme mechanical compliance (100% strain tolerance), a combination difficult to attain with solid-state conductive materials.

The system was further integrated with strain sensors and heaters on a single flexible patch (Figure 3(via–vid)). Two series-connected batteries directly powered a Bluetooth chip and sensing circuits at 3 V, enabling wireless motion data transmission. Gallium-based materials support digital printing for customizable shapes and mass loading, while deep eutectic solvent (DES) technology achieved 98% gallium recovery, addressing e-waste concerns.

#### 3.1.4. Non-Metal Batteries

Non-metal ion batteries are gaining traction due to renewable charge carriers and low cost [60,61]. 3D/2D composite designs are key to overcoming energy density limitations. Yang et al. [62] developed a poly(N-hydroxyethyl acrylamide)/ammonium sulfate/glycerol (PHEA/AS/Gly) ionic hydrogel via photopolymerization and soaking. Its 3D physically cross-linked network, stabilized by hydrogen bonds, exhibited fatigue resistance (<5% conductivity loss after 1000 stretches). Paired with a 2D V_2_O_5_ nanosheet anode and CuFe Prussian blue analog (PBA) cathode, the ammonium-ion battery delivered 68 mAh g^−1^ at −40 °C. The 2D open framework of PBA selectively transported NH^4+^ via size effects, suppressing side reactions and enhancing cycling stability (82% capacity retention after 500 cycles).

In summary, chemical batteries, particularly advanced flexible designs like zinc-ion and lithium-ion systems, provide high energy density and stable power output, making them highly suitable for continuous, long-term epidermal monitoring applications such as uninterrupted ECG or core body temperature tracking. Their ability to function across a wide temperature range further broadens their utility. However, the reliance on pre-stored energy, finite cycle life, potential safety concerns related to electrolyte leakage, and the inherent bulk (even in flexible forms) pose limitations, especially for ultra-thin, disposable, or seamlessly integrated skin electronics. These challenges motivate the exploration of alternative power sources that can harvest energy directly from the body or its immediate environment. Biofuel cells, which directly convert biochemical energy from metabolites into electricity, represent one such promising avenue, offering a pathway to self-powered sensing systems, as will be discussed next.

### 3.2. Biofuel Cell-Powered Epidermal Sensors

Biofuel cells (BFCs) enable integrated sensing and power generation through metabolite-driven mechanisms, making them ideal for dynamic metabolic monitoring [63]. Current limitations in enzyme stability and power density necessitate advancements in enzyme-free catalytic materials. BFCs have achieved breakthroughs in miniaturization, longevity, and precision for non-invasive sensing systems through a “metabolite-driven, self-powered, integrated detection” mechanism. Traditional epidermal sensors rely on external power sources, which not only compromise wearability due to battery bulkiness but also face maintenance challenges from frequent energy replacement. In contrast, BFCs generate electricity by directly oxidizing endogenous metabolites (e.g., ascorbic acid, glucose, ethanol, and lactate) in sweat or interstitial fluid [64,65,66,67]. These endogenous metabolites are continuously replenished through human metabolism, enabling sustained energy production. By dynamically coupling biomarker concentrations with electrochemical signals, BFCs eliminate rigid battery modules while reducing device thickness to submillimeter scales, significantly enhancing wearability [66,68].

Current limitations in enzyme stability and power density necessitate advancements in enzyme-free catalytic materials. Recent breakthroughs in triphase interface engineering demonstrate promising alternatives. Kang et al. [63] developed a wearable lactic acid/O_2_ biofuel cell with air-breathing biocathode using superhydrophobic Pt-deposited electrodes, creating gas-solid–liquid triphase interfaces that enhanced oxygen supply efficiency. This design achieved 1.78 mW cm^−2^ power density through optimized 9:1 cathode/anode ratio, maintaining 94.8% current retention over 10,000 s. The “island-bridge” configuration on flexible substrates enabled 30-day epidermal monitoring while powering heartrate sensors during exercise, exemplifying how 3D interface engineering resolves enzymatic systems’ oxygen diffusion constraints. Similarly, Liu et al. [69] fabricated a high-performance lactate/O_2_ BFC using a 3D interpenetrating network porous structure CNT-membrane, achieved by non-solvent-induced phase separation. This hierarchical porous structure facilitated fast mass-transfer kinetics, uniform enzyme accommodation, and exceptional flexibility, resulting in an impressive power density of 1.6 mW cm^−2^ in artificial sweat with 20 mM lactate and continuous energy harvesting from human sweat for over 36 h, capable of powering integrated circuits. This power density is comparable to state-of-the-art air-breathing BFCs [63], yet Liu et al.’s approach focuses on material architecture for sweat-based applications, potentially offering better mechanical stability and direct skin integration without complex air-cathode designs.

Furthermore, dimension-engineering strategies based on multidimensional nanomaterials endow BFCs with exceptional mechanical robustness, enabling tolerance to over 30% tensile strain or 180° bending deformation [70,71]. This addresses signal drift caused by interfacial delamination or electrode fracture during physical activities. Crucially, the synergistic design of catalytic materials and flexible substrates (e.g., metal nanowires and carbon-based aerogels) optimizes electron transport pathways and biocompatible interfaces, circumventing performance degradation from enzyme deactivation or electrode corrosion in traditional enzymatic sensors [72,73]. This extends continuous monitoring cycles to over 30 days, providing reliable tools for chronic disease management and personalized medicine. The development of novel hydrogel electrolytes also plays a vital role. For instance, Yuan et al. [67] developed self-adhesive wearable hybrid BFCs based on a PVA/succinic anhydride (SAA)-polydopamine (DA) hydrogel. This hydrogel exhibited excellent biofluid absorption and tissue adhesion, enabling the BFC to be directly adhered to human skin to scavenge sweat lactate as fuel, producing a power density of 85.34 µW/cm^2^ and capable of powering a watch.

**Figure 4:**
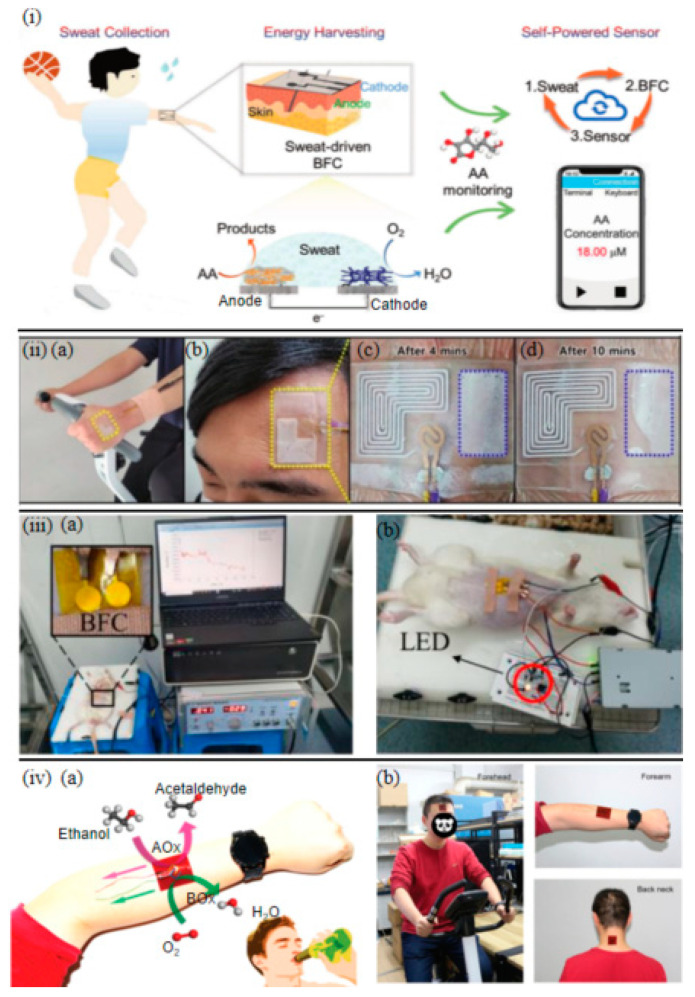
(**i**) Schematic illustration of the metal hydrogel-based integrated w-BFC for sweat collection, energy harvesting, and self-powered sensor [17]. (**iia**) A glucose biofuel cell attached to a subject’s arm. (**iib**) Another glucose biofuel cell attached to a subject’s forehead. (**iic**,**iid**) Progressive activation of a patterned fuel cell after 4/10 min. (**iiia**) Photo of the animal experiment [16]. (**iiib**) Photo of an LED, lit up based on the BFC [1]. (**iva**) Schematics of the flexible and wearable epidermal ethanol BFC for on-body and real-time bioenergy harvesting. (**ivb**) Photographs of a subject wearing the epidermal ethanol BFCs at different skin regions [81].

Ascorbic acid (AA), a critical antioxidant in sweat, exhibits concentration anomalies linked to immune dysfunction and neurodegenerative diseases (e.g., Alzheimer’s disease) [74]. For AA detection, Chen et al. [17] demonstrated the advantages of 1D nanomaterials. They developed a dual hydrogel anode combining 1D gold nanowires (AuNWs) with 2D reduced graphene oxide (rGO). The 1D AuNWs enhanced AA oxidation via surface plasmon resonance, while the 2D rGO conductive network accelerated electron transfer. This synergy achieved a detection sensitivity of 1.0 µM (S/N = 3) and stable power output of 35 µW cm^−2^ at 0.5 mM AA. The integrated wearable BFC (w-BFC) enabled real-time AA monitoring in sweat. Data transmission to user interfaces facilitated health tracking, forming a closed-loop system that integrates sweat collection, energy harvesting, and self-powered sensing (Figure 4i). The 3D cross-linked porous structure of the PtCu bimetallic hydrogel cathode maintained a 0.4 V open-circuit voltage for over 30 days under mechanical deformation, highlighting the reliability of self-powered systems in dynamic monitoring.

Glucose detection is vital for real-time glycemic monitoring to prevent diabetic crises [75,76,77]. Chan Wool Bae et al. [16] constructed a fully stretchable anode using 1D nanoporous gold (NPG), leveraging its 1D channels for direct glucose oxidation without enzyme instability. The cathode employed platinum nanoparticle-decorated 3D micropatterned NPG (PtNPs@NPG), retaining 62.33 mV mM^−1^ sensitivity under 30% tensile strain. A cotton thread-embedded microfluidic channel passively regulated sweat flow via capillary action, enabling continuous 12 h monitoring during intense exercise (Figure 4(iia–iid)). This design demonstrated the adaptability of enzyme-free BFCs in complex physiological environments.

Similarly, Zheng et al. [1] utilized 1D NPG anodes (Figure 4(iiia,iiib)) with directional channels to maintain glucose sensitivity (62.33 mV mM^−1^) under 30% strain. Acetylcholine iontophoresis modulated subcutaneous interstitial fluid (ISF) glucose permeation, while 3D micropatterned PtNPs@NPG cathodes enabled sustained energy generation (Figure 3(iic)). In vivo rat experiments (Figure 4(iic)) demonstrated glucose-driven Light-Emitting Diode (LED) illumination (Figure 4(iid)). Integrating BFCs with display technologies for intuitive readouts is another key advancement. Di et al. [8] constructed a wearable sensor for non-invasive glucose detection from ISF using a screen-printed chip where a BFC drives an electrochromic display. Glucose extracted via reverse iontophoresis fueled the BFC, producing a color change in the display that allowed for visual or smartphone-based RGB value reading.

Visualized sensing based on BFCs extends to other metabolites as well. For example, Wei et al. [78] developed a reusable self-powered electrochromic sensor patch for on-site visualized monitoring of lactic acid in sweat. This patch utilized a lactic acid-fueled BFC to induce a color change in a Prussian blue layer, with a hydrophilic agarose hydrogel and a SiO_2_ hydrophobic film managing sweat collection and elimination. The patch could be regenerated by applying an external voltage, demonstrating its reusability.

2D materials further enhanced sensitivity and flexibility. Qin et al. [65] employed 2D carbon nanotube-reduced graphene oxide (CNTs-rGO) films as bioanode substrates for detecting environmental pollutant dibutyl phthalate (DBP). The layered structure immobilized glucose oxidase (GOD) and 3D Bi_3_Ti_2_O_8_F nanozymes, stabilizing electron transport via π–π stacking. This boosted glucose oxidation current density to 92 µA cm^−2^, achieving a 0.1 nM DBP detection limit and only 8% power density loss at 180° bending, showcasing the synergy of 2D/3D hybrid structures.

Ethanol-powered systems expanded fuel diversity [79,80]. Sun et al. [81] integrated 2D CNTs-rGO with 3D nitrogen-doped hierarchical porous carbon aerogels (3D-NHCAs) to create a flexible ethanol BFC (Figure 4(iva)). The layered conductive network accelerated ethanol oxidation kinetics, yielding 8% power loss at 180° bending. Human trials showed peak power density of 1.01 µW cm^−2^ from drinkers’ sweat during cycling (Figure 4(ivb)).

3D materials resolved long-term energy supply and biocompatibility challenges. Chen et al.’s 3D PtCu hydrogel cathodes tuned d-band centers to optimize oxygen reduction, maintaining catalytic activity with <9% power loss after 30 days. Qin et al.’s 3D Bi_3_Ti_2_O_8_F nanozymes reduced the rate-determining step (RDS) energy of glucose oxidation to 1.13 eV, accelerating response. These designs eliminated external batteries and enabled “detection-powering integration” via metabolite-driven in situ energy harvesting (thickness < 0.5 mm), supporting round-the-clock wearable monitoring.

Biofuel cells (BFCs) thus offer a unique advantage for epidermal sensors by directly utilizing physiological metabolites like glucose, lactate, or ethanol as fuel, enabling integrated sensing and power generation. This approach is particularly promising for monitoring specific biomarkers or metabolic states, potentially offering continuous data streams if metabolite supply and enzyme/catalyst stability are maintained. The recent advancements in material design, such as 3D porous architectures and self-adhesive hydrogels, along with integration with visualization technologies, further enhance their practicality for real-world epidermal applications. However, challenges such as limited power density, degradation of enzymatic activity over time, and fluctuations in metabolite concentrations can affect their long-term continuous operational stability. For applications requiring power beyond what BFCs can consistently provide, or where target analytes are not suitable fuel sources, environmental energy harvesters that scavenge ambient energy present a complementary strategy. These systems, which convert mechanical, thermal, or other forms of environmental energy into electricity, will be explored in the following section.

### 3.3. Environmentally Powered Epidermal Sensors

Environmentally powered systems harvest energy from human motion or ambient sources (e.g., thermal gradients and hydrovoltaic transpiration), offering maintenance-free solutions for low-power sensing [82,83,84,85]. Output instability under environmental fluctuations requires hybrid designs with energy storage units. Environmentally powered systems achieve self-sustained sensing by harvesting energy from human motion, thermal radiation, or humidity gradients, with performance enhancement relying on multidimensional nanomaterials optimizing energy conversion efficiency [86].

**Figure 5:**
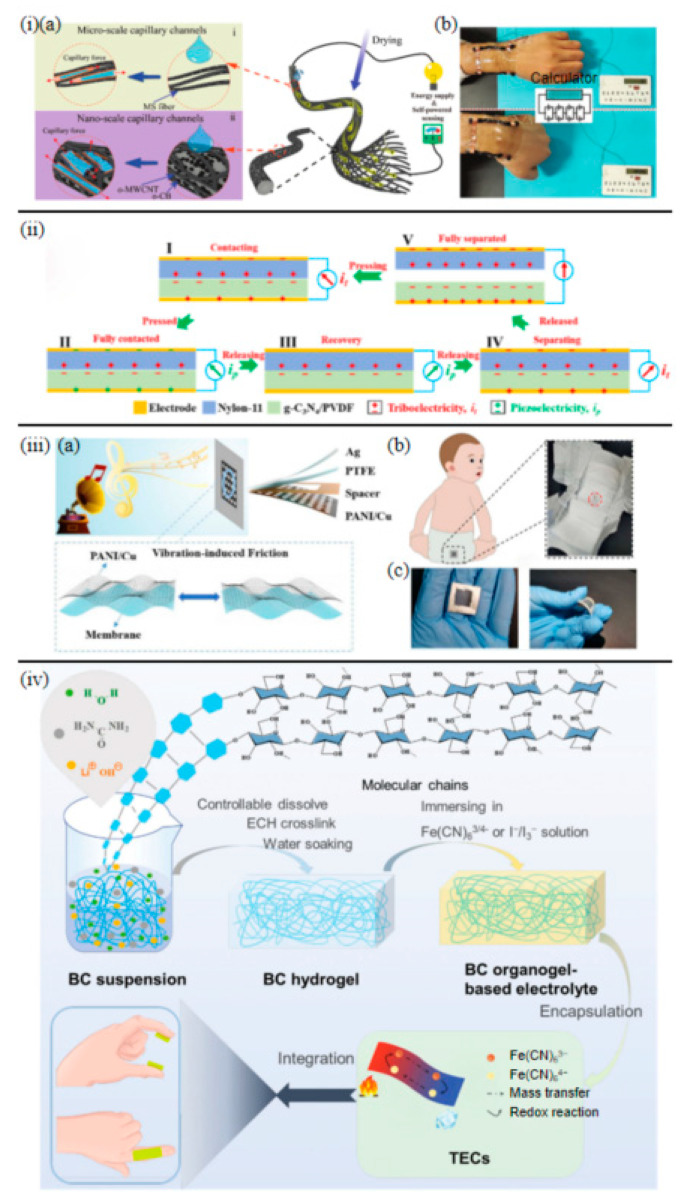
(**ia**) Schematic of multi-scale capillary networks in MS consisting of micro-scale capillary channels and working principle (**ib**) photograph of a portable calculator driven by four groups of power generation units worn at the wrist [87]. (**ii**) Working mechanism of g-C_3_N_4_/PVDF based TENG [88]. (**iiia**) Schematic structure and vibration-induced triboelectric mechanism of the music-driven humidity sensor. (**iiib**) Flexible sensor integration in diapers for real-time urine detection. (**iiic**) Bending stability validation of the TENG-embedded diaper sensor [93]. (**iv**) Preparation diagram, TECs packaging, and conceptual application of BC organogel-based electrolyte [96].

Pioneering the repurposing of discarded mask straps, Luo et al. [87] engineered stretchable fiber-form hydrovoltaic generators (MC-FHPG) through micro-nano capillary networks constructed from 1D oxidized multi-walled carbon nanotubes (o-MWCNTs) and oxidized carbon black (o-CB). As shown in Figure 5(ia), the axial conductivity of 1D carbon nanotubes synergizes with the hierarchical porosity of the fibrous substrate, generating a peak voltage of 0.43 V and energy density of 5.833 mWh cm^−3^ under 0.1 mL water droplet activation. The device maintains stable output under 30% tensile strain and enables joint motion energy harvesting through textile integration, exemplified by a wrist-mounted hydrovoltaic generator powering calculators during bending (Figure 5(ib)). The use of 1D carbon nanomaterials within the fibrous matrix is key to achieving efficient water droplet-induced power generation, offering an alternative to conventional moisture or humidity-based harvesters.

Triboelectric nanogenerators (TENGs) and piezoelectric nanogenerators (PENGs) have garnered significant attention for their ability to convert ambient mechanical energy, particularly human motion, into electrical power for wearable epidermal sensors. Yang et al. [88] embedded 2D graphitic carbon nitride (g-C_3_N_4_) into a PVDF matrix, leveraging its thickness-independent piezoelectric effect to enhance triboelectric output. The fabricated TENGs achieved an open-circuit voltage of 339 V and power density of 0.94 W m^−2^ The 2D lamellar structure of g-C_3_N_4_ creates continuous charge-trapping sites, synergizing with nylon-11 nanofiber positive layers to maintain <5% performance degradation after 5000 cycles (Figure 5ii).

Further advancements in TENG design for epidermal application include the development of ultra-sensitive and stable sensors for physiological monitoring. For instance, Xu et al. [89] developed a self-powered wearable triboelectric sensor utilizing ripple-shaped flexible metal electrodes and electro-spun PVA fiber membranes. This sensor exhibited high sensitivity (6.875 V/kPa) and fast response (7.9 ms), enabling precise detection of subtle physiological signals like the dicrotic pulse wave and demonstrating excellent long-term stability, crucial for continuous cardiovascular health monitoring.

The integration of functional materials to enhance the performance and versatility of PENGs is another active research area. Piezoelectric materials have long been utilized for their energy conversion capabilities in various fields, from industrial sensors for fault detection [90] to a growing range of applications in wearable energy harvesting for epidermal electronics, where they convert biomechanical motion into electrical power. Chen et al. [91] reported high-performance piezoelectric flexible nanogenerators based on graphene oxide (GO) and polydopamine-modified zinc oxide (ZnO) nanoparticles dispersed in a poly(vinylidene fluoride-trifluoroethylene) (P(VDF-TrFE)) matrix. The polydopamine modification improved the dispersion of ZnO and enhanced the piezoelectric properties, while GO contributed to increased electrical output. These PENGs effectively harvested energy from human motion and even from the mechanical impact of bicycle wheels, showcasing their potential for powering wearable devices and smart transportation applications.

Chen et al.’s [91] strategy of using GO and polydopamine-modified ZnO in P(VDF-TrFE) focuses on enhancing the intrinsic piezoelectric properties of the composite material, aiming for higher energy conversion efficiency from mechanical deformation. This material-centric approach differs from TENGs that rely purely on contact electrification and electrostatic induction [91,92]. While PENGs can offer continuous power output during sustained vibration or pressure changes, their output voltage is often lower than that of TENGs, potentially requiring more complex power management circuitry.

Recent advancements demonstrate the potential of hybrid energy harvesting approaches. For example, Qin et al. [92] developed a hybrid triboelectric-piezoelectric smart squirrel cage capable of self-powering and self-sensing for aero-engine condition monitoring. By ingeniously utilizing the supporting structure, this system harvests energy from relative motion and vibration, achieving a maximum power output of 100.0 µW and enabling self-powered sensing of parameters like rotating speed and bearing faults, even powering wireless data transmission for temperature and humidity. While applied to a specific mechanical system, this work highlights the trend towards integrating multiple energy harvesting mechanisms to create robust, multifunctional self-powered systems, a concept highly relevant for advancing complex epidermal electronic devices.

Meanwhile, Peng et al. [93] designed a music-driven humidity sensor based on 2D polytetrafluoroethylene (PTFE)/polyaniline (PANI) heterojunctions. Soundwave-induced resonance triggers periodic contact-separation between triboelectric materials with opposite polarities, generating 0.78 nA current (Figure 5(iiia)). Embedded in diapers (Figure 5(iiib)), this flexible device (Figure 5(iiic)) exhibits linear signal attenuation with humidity (sensitivity −0.52% RH^−1^), enabling battery-free real-time monitoring of infant urination. Beyond direct power generation, TENGs can also be integrated with other functionalities. For example, Zheng et al. [94] developed self-regulating heating and self-powered flexible fiber fabrics based on azopolymer-nylon composites. These fabrics not only exhibited triboelectric power generation from friction but also demonstrated a photo-induced solid–liquid transition for self-regulating heating at low temperatures, highlighting a multifunctional approach for wearable devices in extreme environments.

Recent advances in solar-powered wearables demonstrate efficient energy autonomy through rational photovoltaic-battery integration [95]. Chong et al. [3] developed a solar–battery hybrid electrocardiogram (ECG) monitoring system using 1D polycrystalline silicon photovoltaic arrays (95 × 95 mm^2^, 6 V/3 W) and 3D stacked Li-ion batteries (3.7 V/2400 mAh). The 1D nanowire-structured solar cells achieved 5–11 h outdoor charging cycles (621.8 W m^−2^ irradiance) with 48–60 h device runtime, while 2D carbon nanotube networks in circuit design minimized energy loss (<20%). This architecture maintains stable operation through circadian rhythm-aligned energy management-daytime solar harvesting (5.5–6.2 V output) synergizing with nighttime battery buffering, establishing a prototype for multidimensional nanomaterial-integrated epidermal electronics.

Advancing thermal energy conversion, Li et al. [96] developed bacterial cellulose (BC) organogel thermocells using propylene glycol-induced K_4_Fe(CN)_6_ crystallization to establish 3D concentration gradients, boosting the Seebeck coefficient to 2.30 mV K^−1^. 3D nanofiber network of bacterial cellulose facilitates rapid Fe(CN)_6_/_4_^−^ diffusion, while 20 vol% propylene glycol enhances solvation effects, achieving 0.8 mA cm^−2^ current density under body heat gradients (Figure 5iv).

Sun et al. [97] created strain-resistant thermal sensors using CNT/MXene hybrids and Si_3_N_4_ nanoparticle-reinforced paper substrates. The 2D conductive layers of MXene and the 1D conductive pathways of CNTs synergistically form 3D percolation networks, which maintain a thermal sensitivity of −0.52% °C^−1^ even after 2000 bending cycles.

Environmental energy harvesters, including TENGs, piezoelectric, thermoelectric, and hydrovoltaic generators, demonstrate considerable potential for powering epidermal sensors by converting readily available ambient energy—such as body motion, thermal gradients, or moisture—into electrical power. These are particularly suited for low-power, intermittent sensing scenarios, and in some cases, the harvesting mechanism itself can be part of the sensing modality (e.g., TENGs for motion sensing or self-powered physiological monitoring). While offering maintenance-free operation, their output is often inherently sporadic and dependent on environmental conditions, potentially requiring integration with energy storage elements like supercapacitors or thin-film batteries for more stable, continuous operation. The development of multifunctional materials that combine energy harvesting with other features like self-heating further expands their applicability in diverse wearable systems. For applications demanding on-demand power without on-board energy storage or harvesting complexity, or for ultra-thin disposable devices, wireless power transfer technologies offer a compelling alternative, which we will now examine.

### 3.4. Battery-Free Epidermal Sensors with Wireless External Power Supply

Wireless power technologies eliminate energy storage units through near-field coupling, enabling ultra-thin disposable sensors [98,99,100,101]. Limited transmission distance and antenna deformation compatibility demand further optimization of flexible antenna materials. Wireless external power technologies, including NFC and radiofrequency (RF) energy harvesting, enable battery-free sensing systems [102,103,104]. Their performance enhancements relies on breakthroughs in multidimensional nanomaterials for energy conversion efficiency, flexible antenna design, and system integration [105].

**Figure 6:**
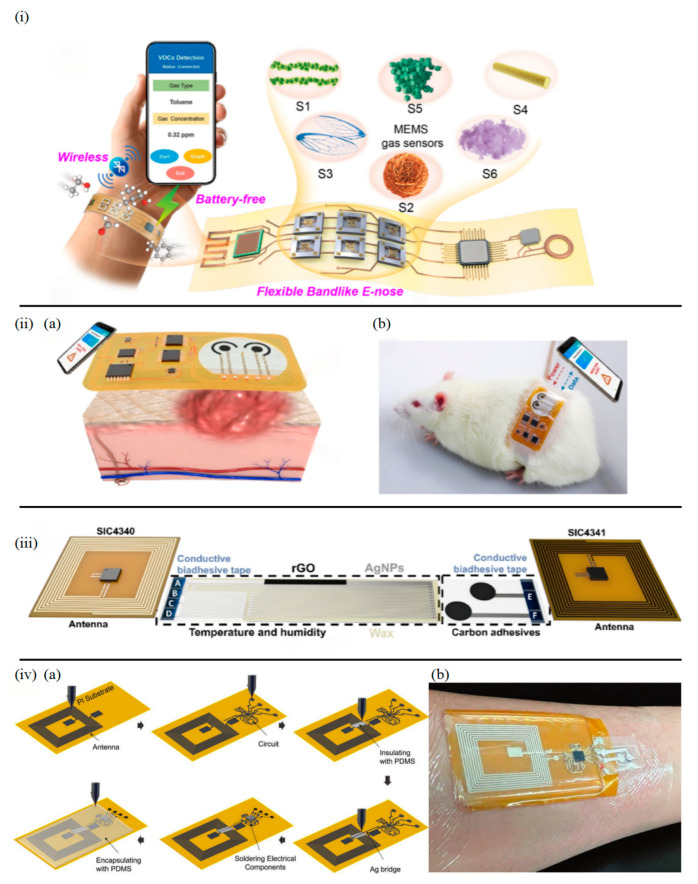
(**i**) Scheme illustration of the battery-free, wireless, flexible bandlike e-nose system design based on MEMS gas sensors [106]. (**iia**) Schematic of the smart bandage system for in situ bacterial virulence factors detection. (**iib**) Photograph of a freely moving rat with the smart bandage adhered on the wound site [18]. (**iii**) Schematic of the epidermal sensor network for heat stroke detection [12]. (**iva**) the WB2F3D sensor system fabrication steps on skin-like SEBS substrate. (**ivb**) Photo of the WB2F3D sensor system attached to the human forearm for the real-time and wireless pH measurement of pH changes [19].

1D nanostructures demonstrate unique advantages in flexible antenna and energy harvester design. Yang et al. [106] developed a stretchable NFC antenna using silver nanowires (AgNWs), where the optimized spiral structure achieved an inductance of 2.75 µH through directional alignment of 1D nanowires. Coupled with a silicon-based NFC chip, this e-nose system achieved precise resonance at 13.56 MHz (Figure 6i). The system exhibited detection limits as low as 200 ppb for Volatile Organic Compounds (VOCs) like formaldehyde and toluene, maintaining 98% signal stability in vehicular environments, validating the reliability of 1D materials in complex electromagnetic environments.

2D MXene materials significantly enhance sensor sensitivity and stability through their high conductivity and surface modifiability. Shi et al. [18] created an MXene-based smart bandage system (Figure 6(iia)) featuring Ti_3_C_2_T_x_ MXene/gold nanoparticles/peptide (Fc-LPETGC) composite-modified electrodes. This system achieved dual detection of Staphylococcus aureus sortase A (1 pg mL^−1^ detection limit) and Pseudomonas aeruginosa pyocyanin (1 µM detection limit). The 2D layered MXene structure enhanced electron mobility to 8 × 10^5^ m s^−1^, while the NFC module (13.56 MHz ± 5% resonant frequency) enabled stable energy harvesting within 30 mm distance, successfully monitoring wound infection dynamics in animal models (Figure 6(iib)).

Maroli et al. [12] optimized 2D material printing processes by directly fabricating silver nanoparticle (AgNP) antennas and sensor arrays on bioinspired adhesive membranes (Figure 5iii). MXene-modified temperature/humidity sensors showed 3× sensitivity enhancement, while muscle contraction detection electrodes enabled smartphone-based wireless monitoring of heat stroke risk factors through NFC. This 2D printing strategy achieved an antenna quality factor of 32 with extended 5 cm transmission distance.

3D printing technology enables structural customization and functional integration for wireless sensors [107]. NajafiKhoshnoo et al. [19] pioneered multimaterial 3D printing to construct a wearable wireless pH sensing system on skin-like flexible substrates (Figure 6iv). Through 3D multilayer printing of silver nanoparticles, PANI, and Ag/AgCl (Figure 6(iva)), the sensor demonstrated high sensitivity (≈51.76 mV pH^−1^) across pH 3.0–10.0 and mechanical stability (>90% performance retention after 30% bending cycles). The integrated 3D spiral NFC antenna and flexible circuitry enabled 2 cm-distance wireless powering and real-time data transmission, as shown in Figure 6(ivb) depicting an integrated, miniaturized, modular, wearable, battery-free, biocompatible, flexible, 3D-printed (WB_2_F_3_D) sensor operation on human forearm.

3D nanocellulose networks provide sustainable solutions for high-loading electrode design. Li et al. [108] developed flexible self-supported electrodes using NFC 3D porous frameworks coupled with LFP/graphene (GR) conductive networks. At 45 wt% LFP loading, surface resistance decreased by 93%, achieving >90% capacity retention after 770 cycles at 5 C rate, demonstrating the potential of 3D bio-based materials in integrated wireless energy storage-sensing systems.

In conclusion, this section has reviewed four distinct power supply strategies for epidermal sensors: chemical batteries, biofuel cells, environmental energy harvesters, and wireless power transfer. Each strategy presents a unique set of advantages and limitations concerning energy density, operational lifetime, form factor, biocompatibility, and suitability for different sensing modalities and application scenarios. Chemical batteries excel in continuous, high-power demand applications but face challenges in miniaturization and safety. Biofuel cells offer an elegant solution for self-powered biomarker sensing by leveraging physiological fuels, though enzyme stability and power output remain key hurdles. Environmental energy harvesters provide pathways to maintenance-free, autonomous operation by scavenging ambient energy, ideal for low-power or event-driven sensing, but often with intermittent power delivery. Finally, wireless power transfer enables ultra-thin, battery-less designs for on-demand operation, constrained primarily by transmission distance and efficiency. The optimal choice of power system is therefore highly dependent on the specific requirements of the epidermal sensor application, often necessitating a hybrid approach or careful co-design of the sensor and its power source to achieve desired performance and functionality.

## 4. Conclusions and Perspective

This review summarizes recent advancements in power supply technologies for epidermal sensors, categorized into four approaches: chemical fuel cells, biofuel cells, environmental energy harvesting, and wireless power transfer, with application examples detailed in Table 1, Table 2 and Table 3. A systematic comparison of these technologies, including their advantages, limitations, compatible sensor types, and operational continuity, is provided in Table 4. While each strategy offers distinct advantages, significant challenges remain in achieving an optimal balance of energy density, flexibility, long-term stability, biocompatibility, and seamless integration for diverse epidermal applications. Overcoming these hurdles is critical for realizing the full potential of next-generation epidermal electronics.

With the rapid development of epidermal electronics in health monitoring, motion sensing, and environmental detection, constructing efficient, stable, and sustainable energy supply systems has become a core challenge for overcoming current technological bottlenecks. Future research must synergistically advance precise matching of energy supply methods with sensing scenarios, multidimensional material innovations, and intelligent closed-loop system design to propel epidermal sensors towards greener, smarter, and more practical applications.

- Scenario-Specific Energy-Sensing Coupling: Future epidermal power systems will transcend simple energy provision, evolving towards intelligent scenario-specific energy-sensing coupling. This paradigm shift necessitates a dynamic alignment between energy sources and the specific demands of the sensing application, moving beyond one-size-fits-all solutions. For instance, health monitoring sensors reliant on biomolecules (e.g., glucose and lactate detection), may primarily utilize biofuel cells (BFCs) powered by target analytes supplemented by triboelectric nanogenerators (TENGs) harvesting motional energy and integrated with 3D porous carbon aerogel-based supercapacitors for transient energy storage and stabilization. Composite electrodes, such as MXene/carbon nanotube composite electrodes can enhance BFC catalytic stability (enzyme activity retention > 90%), while bioinspired microstructured TENGs (output density > 5 µW cm^−2^) improve mechanical strain sensitivity. Conversely, for motion monitoring sensors (e.g., strain and pressure detection), hybrid systems combining environmental energy (TENGs, piezoelectric materials) with miniaturized zinc-ion batteries are advantageous. Ag nanowire/PVDF composite TENGs efficiently convert mechanical energy, while fatigue-resistant 3D interlocking electrode modules (e.g., PAAM/CMC/LiCl hydrogel batteries) mitigate intermittent power supply caused by sporadic movements. In low-power wireless gas sensing scenarios (e.g., NO_2_ and VOCs detection) pairing near-field communication (NFC) with photothermal energy harvesting (e.g., MXene/graphene heterojunction photothermal films integrated with flexible perovskite solar cells) can enables continuous environmental. These hybrid approaches, potentially governed by intelligent power management circuits, aim not only to ensure continuous operation but also to enhance sensor sensitivity or enable new sensing modalities by leveraging the unique characteristics of each power source in synergy, marking a key direction towards truly autonomous and highly efficient epidermal devices.
- Multidimensional Material Engineering and Bioinspired Structural Innovations: Breakthroughs in multidimensional material engineering and bioinspired structural innovations are fundamental to overcoming the intrinsic limitations of current epidermal power systems in terms of performance, durability, and biocompatibility. For instance, meticulous 1D/2D heterojunction dynamic or 3D porous architectures (e.g., MXene-carbon composites) enhance ion/electron transport and mechanical resilience. Bioinspired dynamic interfaces, like self-healing poly(3,4-ethylenedioxythiophene) polystyrene sulfonate (PEDOT:PSS) hydrogels with liquid metals, improve reliability under mechanical stress. Critically, a shift towards sustainable, biodegradable materials (e.g., silk, chitosan, and bacterial cellulose) is essential to mitigate e-waste from disposable epidermal devices, with concepts like enzymatically degradable pH-responsive batteries showing a promising path.
- Intelligent Closed-Loop Systems: The evolution towards intelligent closed-loop systems will transform epidermal electronics from passive data collectors into autonomous entities capable of real-time adaptation and optimized performance. For example, 2D g-C_3_N_4_/PANI heterojunctions can concurrently monitor sweat pH/ion concentrations and power micro-supercapacitors, forming self-sustaining energy chains. The integration of Artificial Intelligence (AI) will be crucial for future epidermal power systems. Specifically, machine learning algorithms, such as Long Short-Term Memory (LSTM) networks, can enable predictive power management. This allows for dynamic optimization of energy allocation based on user activity and environmental conditions. Furthermore, technologies like flexible memristors offer pathways for adaptive energy regulation. Ultimately, these AI-driven approaches can significantly extend system runtime and enhance robustness against energy fluctuations.
- Scalable Manufacturing and Environmental Resilience: Transitioning advanced epidermal power systems from laboratory prototypes to widespread practical applications hinges on breakthroughs in scalable manufacturing and extreme-environment tolerance are imperative. Advances in printed electronics (e.g., roll-to-roll 3D printing) allow one-step fabrication of MXene/ZnO nano-ink-based microbatteries and gas sensor arrays (line width < 50 µm), drastically reducing costs. For harsh environments (e.g., high temperature/humidity), SnS_2_-based encapsulation combined with ionic liquid gel electrolytes ensures stable operation (>1000 h at 85 °C/95% RH), expanding applications in tropical healthcare and industrial monitoring.

In summary, next-generation epidermal sensor energy systems will exhibit scenario-specific customization, energy diversity, and material intelligence. By precisely aligning energy solutions with sensing demands, innovating multidimensional materials, and integrating AI with flexible electronics, these systems will overcome existing limitations, transitioning from lab prototypes to scalable applications. This progress promises revolutionary advancements in smart healthcare, human–machine interfaces, and environmental monitoring.

## Figures and Tables

**Table 1:** Examples of chemically powered epidermal sensors. PAAM/CMC/LiCl: polyacrylamide/carboxymethyl chitosan/LiCl. rGO: reduced graphene oxide. LBG: locust bean gum. GO: graphene oxide. PVDF-HFP: polyvinylidene fluoride-co-hexafluoropropylene. P(BA-co-EGDMA): poly(butyl acrylate-co-ethylene glycol dimethacrylate). HNT: halloysite nanotubes. EG-LiFePO_4_: electrochemically exfoliated graphene–lithium iron phosphate. EG-Li_4_Ti_5_O_12_: electrochemically exfoliated graphene–lithium titanate. AgNWs: silver nanowires. PDMS: polydimethylsiloxane. PHEA/AS/Gly: poly(N-hydroxyethyl acrylamide)/ammonium sulfate/glycerol. Ga-CB-SIS: gallium–carbon black-styrene/isoprene block copolymer.

	Power Supply Type	Material Innovation	Modification/Functionalization/Fabrication	Specialized Properties	Voltage	Capacity /Specific Capacity/Power Density	Motion Detection	Ref
Chemically Powered Epidermal Sensors	Zn//MnO_2_ Battery	rGO; Locust bean gum	MnO_2_/rGO composite; LBG-based hydrogel	-	1.4 V	2.72 mAh	Large-range human motion	[36]
	Zn//MnO_2_ Battery	PAAM/CMC/LiCl	Prepared via one-step UV radical polymerization	Anti-freezing capability; self-healing	1.4 V	214.2 mAh g^−1^	Human motion detection	[44]
	Cu/GO[Ca]/Zn battery	GO	Ca^2+^ intercalation in GO layers		0.77 V	1.93 mW cm^−2^	Human motion detection; Morse code generation via finger bending	[38]
	LFP//Li battery	PVDF-HFP; P(BA-co-EGDMA); HNT	UV-curing enables rapid processing; 3D polymer network enhances mechanical properties	Effective lithium dendrite suppression	3.2~3.8 V	123.8 mAh g^−1^	-	[49]
	LTO//LFP battery	Exfoliated graphene	EG-LiFePO_4_ nanocomposite; EG-Li_4_Ti_5_O_12_ nanocomposite	Withstand bending/folding/twisting	2 V	137 mAh g^−1^	-	[52]
	LTO//LFP battery	AgNWs	AgNWs/PDMS Stretchable Current Collectors		2 V	108 mAh g^−1^	-	[56]
	LFP//C battery	PVDF-HFP; Nano-graphite; CNT	LiFePO_4_/C nanoparticles synthesized via solid-state method with carbon coating; sequential electrospinning	Semi-transparency	3.35 V	140 mAh g^−1^	-	[57]
	AIB/NH^4+^	PHEA/AS/Gly		Anti-freezing capability	0.8–1.2 V	42.5 mAh g^−1^	Human motion detection	[62]
	Ag-Ga battery	Ga-CB-SIS	3D-printed	self-healing; recyclability	1.6–1.8 V	26.86 mAh cm^−2^	Monitor finger bending gestures	[15]

**Table 2:** Examples of biofuel cell-powered epidermal sensor. CP: carbon paper; PtCu NPs: platinum-copper nanoparticles. Au-rGO: gold-reduced graphene oxide. NPG: nanoporous gold. PtNPs: platinum nanoparticles. PDA: polydopamine. CNTs-rGO: carbon nanotubes-reduced graphene oxide. Bi_3_Ti_2_O_8_F: bismuth titanate oxyfluoride (chemical formula, no expansion needed). AuNPs: gold nanoparticles. EDC/NHS: 1-ethyl-3-(3-dimethylaminopropyl)carbodiimide/N-hydroxysuccinimide. BSA: bovine serum albumin. DBP: dibutyl phthalate. 3D-NHCAs: three-dimensional nitrogen-doped hierarchical carbon aerogels.

	Metabolite	Material Innovation	Modification/Functionalization/Fabrication	Voltage	Capacity /Specific Capacity/Power Density	Biomarker Detection	Ref
Biofuel Cell-Powered Epidermal Sensors	Lactic acid	Pt-deposited CP; BOD/CNT	Pt electrodeposition creating triphase interface; BOD/CNT/Nafion membrane casting	0.75 V	1.78 mW cm^−2^	Heart rate sensing integration	[63]
	Lactic acid	CNT-membrane (3D porous)	3D interpenetrating network porous CNT-membrane bioanode (via NIPS); Air cathode	OCV > 0.84 V (in 20 mM lactate)	1.6 mW cm^−2^ (at 20 mM lactate)	Energy harvesting from sweat; powering bluetooth	[69]
	Lactic acid	PVA/SAA-DA hydrogel	Self-adhesive PVA/SAA-DA hydrogel electrolyte based hybrid BFC (HBFC)	OCV 0.57 V	85.34 µW cm^−2^	Powered by human sweat; can power a watch	[67]
	Ascorbic acid	PtCu NPs; Au-rGO	PtCu bimetallic hydrogel; Au-rGO dual hydrogel	0.4 V	35 µW cm^−2^	Ascorbic acid detection	[17]
	Glucose	NPG	PtNPs@NPG	62.33 mV M^−1^	2.512 µW cm^−2^ M^−1^	Glucose detection	[16]
	Glucose	PtNPs	Drop-casting enzyme/PtNPs/chitosan mixtures	0.151 V	1.9 μW	Glucose detection	[1]
	Glucose	Screen-printed chip; Agarose gel; PDA-CNTs	BFC-driven electrochromic display; PDA-CNTs doped agarose gel electrolyte; reverse iontophoresis for glucose extraction	——	——	Glucose detection (visualized by color change/RGB)	[8]
	Lactic acid	Agarose hydrogel; SiO_2_ hydrophobic film	Hydrophilic agarose hydrogel and SiO_2_ hydrophobic film for sweat management	OCV 0.3 V	5.2 µW cm^−2^	On-site visualized monitoring of lactic acid	[78]
	Glucose	CNTs-rGO; Bi_3_Ti_2_O_8_F; AuNPs	Bi_3_Ti_2_O_8_F immobilized on CNTs-rGO via chitosan; AuNPs modified CNTs-rGO activated by EDC/NHS for aptamer binding, blocked with BSA	0.3 V	300 µW cm^−2^	DBP	[65]
	Ethanol	3D-NHCAs	Developed 3D coral-like nitrogen-doped hierarchical micro-mesoporous carbon aerogel (3D-NHCAs)	0.39 V	1.9 µW cm^−2^	Ethanol	[81]

**Table 3:** Examples of Environmentally powered epidermal sensors. HPG: hydrovoltaic power generator. TENG: triboelectric nanogenerator. g-C_3_N_4_: graphitic carbon nitride. PVDF: polyvinylidene fluoride. PTEF: polytetrafluoroethylene. PANI: polyaniline. Nylon fabrics: NFs. TECs: thermoelectric cells. K_4_Fe(CN)_6_: potassium ferrocyanide. K_3_Fe(CN)_6_: potassium ferricyanide. CNT: carbon nanotube. MXene: transition metal carbide/nitride. Si_3_N_4_: silicon nitride. PDMS: polydimethylsiloxane.

	Power Supply Type	Material Innovation	Modification/Functionalization/Fabrication	Voltage/OCV	Capacity/Specific Capacity/Power Density	Detection Type	Ref
Environmentally Powered Epidermal Sensors	HPG	Mask straps	Dip-coating method	0.43 V	5.833 mWh cm^−3^	Strain detection	[87]
	TENG	g-C_3_N_4_	Composite of two-dimensional graphitic carbon nitride (g-C_3_N_4_) and PVDF	339 V	0.94 W m^−2^	Human motion and complex gestures detection	[88]
	TENG	Ripple-shaped Ag electrodes; Electro-spun PVA fiber	Single-electrode mode flexible sensor with 3D interconnected porous PVA fiber membrane	——	——	Pulse wave monitoring; cardiovascular health	[89]
	PENG	GO; Polydopamine-modified ZnO; P(VDF-TrFE)	GO-PDA@ZnO/P(VDF-TrFE) piezoelectric nanohybrid films	Up to 48.8 V (4 units in series from bicycle)	——	Human motion energy capture; bicycle nanoenergy harvesting	[91]
	TENG	PTEF; PANI	Oxygen plasma treatment	-	23.7 mW	Humidity detection	[93]
	TENG and photothermal	Azopolymer (PAzo9:1-co-PS);	PAzo9:1-co-PS@NF composite for self-regulating heating and TENG power generation	170 V (−20 °C to 25 °C)	70.6 kJ kg^−1^	self-regulating heating; self-powered flexible fiber fabrics for low temp	[94]
	Solar–battery	1D poly-Si PV arrays; 3D Li-ion stack	1D nanowire-structured solar cells	5.5–6.2 V	2400 mAh	ECG monitoring	[3]
	TECs	Bacterial Cellulose; K_4_Fe(CN)_6_/K_3_Fe(CN)_6_	Bacterial cellulose organogel; propylene glycol modification	0.076 mV (40 °C)	104.2 mW m^−2^ (40 °C)	Strain detection	[96]
	TECs	CNT; MXene; Si_3_N_4_	Hybrid material of CNTs and MXene; flexible paper/PDMS/Si_3_N_4_ composite substrate	-	-	Temperature detection	[97]

**Table 4:** Comparison of chemically powered, biofuel cell-powered, environmentally powered, and battery-free wireless systems for epidermal sensors: performance trade-offs, sensor compatibility, and operational sustainability.

Power Supply Type	Advantages	Limitations	Applicable Sensor Types	Power Continuity	Ref
Chemically Powered	High energy density (e.g., Zn-ion: 214.2 mAh g^−1^), long-term stability, wide temperature tolerance (−20 °C to 200 °C)	Risk of electrolyte leakage, dendrite formation (limited cycle life), insufficient mechanical flexibility	ECG, SpO_2_ sensing, motion monitoring	Continuous (finite lifespan)	[36,44,52,56]
Biofuel Cell-Powered	Self-powered (metabolite-driven), ultra-thin (<0.5 mm), integrated sensing-power generation	Output dependent on metabolite fluctuations (e.g., glucose), enzyme activity degradation (requires enzyme-free catalysts)	Glucose, lactate, ascorbic acid detection, heart rate sensing integration	Continuous (dynamic fluctuations)	[1,17,63,65,81]
Environmentally Powered	Maintenance-free (e.g., TENGs), suitable for low-power intermittent sensing	Unstable output (environment dependent), low energy density (e.g., TENG: 0.94 W m^−2^)	Motion, temperature, humidity, pressure sensing	Intermittent (environment dependent)	[87,88,93,96,97]
Battery-Free Wireless	Ultra-thin wearables, no energy storage (ideal for disposable patches)	Short transmission distance (≤5 cm), antenna deformation sensitivity, efficiency limitations organogel; propylene glycol modifica-tion	Gas (VOCs), short-term wound monitoring	On-demand (requires external transmitter)	[12,18,19,106,108]

## Data Availability

Data will be made available on request.
